# Brain single cell transcriptomic profiles in episodic memory phenotypes associated with temporal lobe epilepsy

**DOI:** 10.1038/s41525-022-00339-4

**Published:** 2022-11-29

**Authors:** Robyn M. Busch, Lamis Yehia, Bo Hu, Melissa Goldman, Bruce P. Hermann, Imad M. Najm, Steven A. McCarroll, Charis Eng

**Affiliations:** 1 Epilepsy Center, Neurological Institute, Cleveland Clinic, Cleveland, OH USA; 2 Department of Neurology, Neurological Institute, Cleveland Clinic, Cleveland, OH USA; 3 Genomic Medicine Institute, Lerner Research Institute, Cleveland Clinic, Cleveland, OH USA; 4 Department of Quantitative Health Sciences, Lerner Research Institute, Cleveland Clinic, Cleveland, OH USA; 5 Department of Genetics, Harvard Medical School, Boston, MA USA; 6 Stanley Center for Psychiatric Research, Broad Institute of MIT and Harvard, Cambridge, MA USA; 7 Taussig Cancer Institute, Cleveland Clinic, Cleveland, OH USA; 8 Department of Genetics and Genome Sciences, Case Western Reserve University School of Medicine, Cleveland, OH USA

**Keywords:** Epilepsy, Gene expression, Genetics research

## Abstract

Memory dysfunction is prevalent in temporal lobe epilepsy (TLE), but little is known about the underlying molecular etiologies. Single-nucleus RNA sequencing technology was used to examine differences in cellular heterogeneity among left (language-dominant) temporal neocortical tissues from patients with TLE with (*n* = 4) or without (*n* = 2) impairment in verbal episodic memory. We observed marked cell heterogeneity between memory phenotypes and identified numerous differentially expressed genes across all brain cell types. The most notable differences were observed in glutamatergic (excitatory) and GABAergic (inhibitory) neurons with an overrepresentation of genes associated with long-term potentiation, long-term depression, and MAPK signaling, processes known to be essential for episodic memory formation.

## Introduction

Temporal lobe epilepsy (TLE) is the most common type of focal epilepsy and is associated with high risk for memory deficits, particularly in those whose seizures do not respond to medication^1^. Importantly, patients report memory difficulties to be among the most concerning aspects of their condition, second only to unexpected seizures and driving restrictions^2^. While a host of demographic and disease-related variables have been associated with episodic memory dysfunction in TLE^3–7^, a substantial proportion of memory performance variance remains unexplained. Significant efforts have been expended to understand the *molecular basis* of memory dysfunction in numerous disorders, particularly Alzheimer’s disease. Yet, by comparison, very little is known about the biological underpinnings of memory dysfunction in epilepsy^8^.

We have recently demonstrated, using classical bulk RNA sequencing, that genes associated with neurological functions are underexpressed in the temporal neocortex of TLE patients with impaired memory compared to those with intact memory, and implicated are genes involved in the pathogenesis of neurodegenerative disorders (e.g., *APOE*, *APP, MAPT*) in memory impairment in TLE^9^. To further understand the molecular basis of memory impairment in TLE, the current study used single-nucleus RNA-Seq (snRNA-Seq) to determine whether specific cell types within the temporal neocortex contribute to memory outcomes in TLE.

## Results

**Fig. 1:**
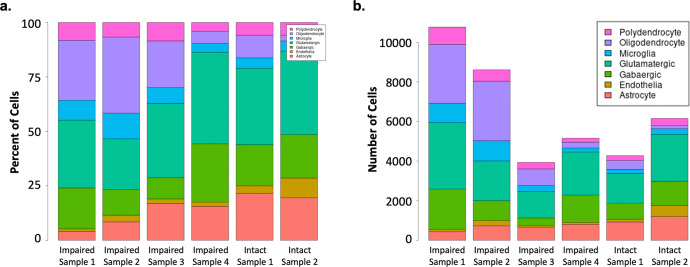
Histogram demonstrating clusters of nuclei by cell type and by patient. Plots of cells from temporal lobe tissue of individual patients with and without memory impairment by (**a**) percent and (**b**) number of each cell type.

The two memory groups were well-matched on all demographic and disease-associated variables *(*Table 1). We observed marked cell heterogeneity between memory phenotypes (Fig. 1 and Supplementary Figure 1) and identified numerous differentially expressed genes across all brain cell types (Supplementary Tables 1 and 2a-g). The most notable differences were observed in glutamatergic (excitatory) neurons (5826 DEGs, *P*_*adj*_ < 0.05; 325 DEGs with log2FC < −1 or >1) followed by GABAergic (inhibitory) neurons (3764 DEGs, *P*_*adj*_ < 0.05; 123 DEGs with log2FC < −1 or >1).

**Table 1:** Demographic and epilepsy-related data for study patients.

	Intact Memory (*n* = 2)	Impaired Memory (*n* = 4)	*P*
	Mean (SD)	Mean (SD)	
Age	47.00 (8.49)	37.75 (9.43)	0.310
Education	15.00 (1.41)	12.00 (0.96)	0.076
Age at Seizure Onset	35.50 (19.09)	22.00 (10.90)	0.309
Duration of Epilepsy (years)	11.50 (10.61)	15.75 (7.27)	0.583
Full Scale IQ	95.00 (1.41)	85.00 (9.85)	0.268
Mean Verbal Delayed Memory	95.75 (6.72)	57.88 (3.79)	<0.001
	*Number (%)*	*Number* (*%)*	
Sex (Female)	1 (50%)	2 (50%)	–
Etiology (MTS)	1 (50%)	2 (50%)	–

*SD* standard deviation; *MTS* mesial temporal sclerosis.

We first implemented a wide net approach to identify biologically relevant pathways using all DEGs with *P*_*adj*_ < 0.05. Pathway analysis using KEGG revealed an overrepresentation of genes associated with long-term potentiation (glutamatergic - *P*_*adj*_ = 2.28 × 10^−6^; GABAergic - *P*_*adj*_ = 0.0015), long-term depression (glutamatergic - *P*_*adj*_ = 1.16 × 10^−5^; GABAergic - *P*_*adj*_ = 0.011), and MAPK signaling (glutamatergic - *P*_*adj*_ = 0.002; GABAergic - *P*_*adj*_ = 0.041), processes known to be essential for episodic memory formation (Supplementary Tables 3a-3e). Interestingly, the cell adhesion molecule (CAM) pathway was a significant pathway hit for every cell type examined between the two memory groups, except for endothelial and microglial cells where no significant differences were identified (Supplementary Tables 3a-3e).

**Fig. 2:**
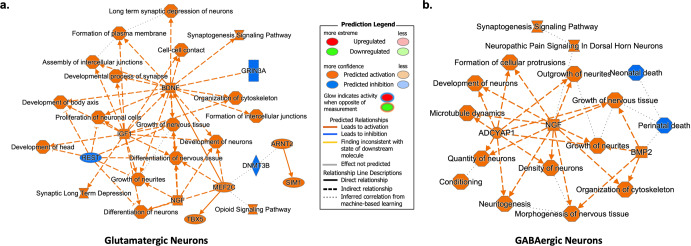
Pathway enrichment analysis of differentially expressed genes from glutamatergic and GABAergic neurons. A machine learning algorithm implemented through Ingenuity Pathway Analysis (IPA) agnostically identifies pathways and molecules relevant to memory function in (**a**) glutamatergic and (**b**) GABAergic neurons. Note that the legend may include predicted events not observed within the constructed networks.

**Fig. 3:**
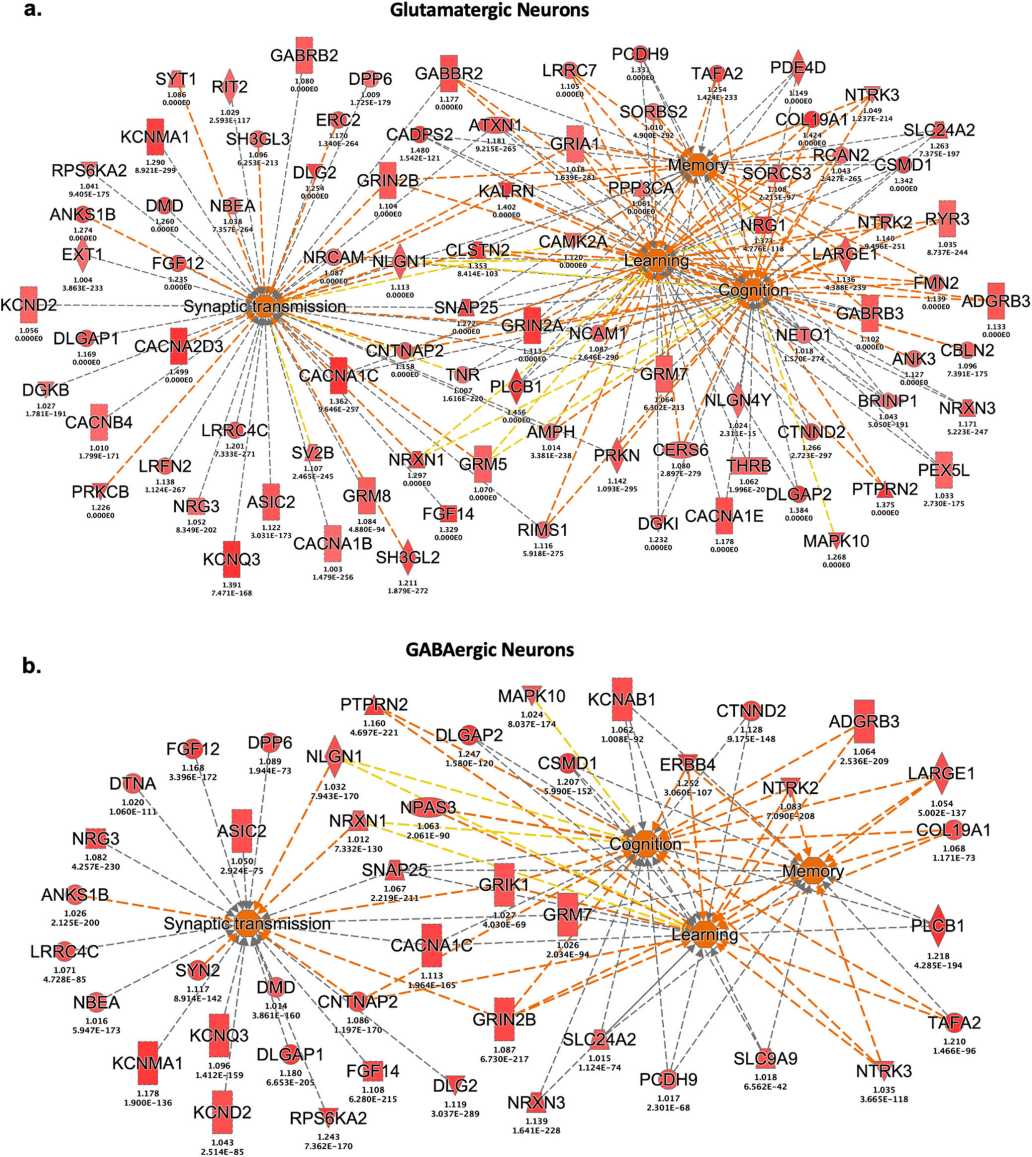
Diseases and functions associated with the differentially expressed genes from glutamatergic and GABAergic neurons. Differentially expressed genes between brains from patients with and without memory impairment converge on memory-related processes as predicted through IPA in both (**a**) glutamatergic and (**b**) GABAergic neurons.

Next, we implemented a more stringent approach to identify biologically relevant pathways using all DEGs with *P*_*adj*_ < 0.05 and with log2FC < −1 or >1. For this analysis, we used Ingenuity Pathway Analysis (IPA), which provides a more comprehensive knowledge base compared to KEGG. Pathway enrichment analysis using IPA, and the more stringent criteria for inclusion of DEGs, corroborated our observations using KEGG (Table 2 and Supplementary Tables 4a-4b). ‘Synaptogenesis’ was identified as the top pathway for both neuronal cell types (glutamatergic - *P*_*adj*_ = 5.3 × 10^−13^; GABAergic - *P*_*adj*_ = 2.2 × 10^−6^). Relatedly, IPA identified multiple networks connecting the synaptogenesis signaling pathways to other functions relevant to neuronal homeostasis and memory pathobiology (Fig. 2). Relevant to our memory phenotype, examination of diseases and functions through IPA yielded the following: ‘memory,’ ‘learning’, ‘cognition,’ ‘long-term potentiation,’ ‘synaptic depression’ and ‘synaptic transmission’ (Fig. 3 and Supplementary Tables 5a-5b). In contrast to the overrepresentation of the cell adhesion molecule (CAM) pathway following the KEGG analyses, we only identified gap junction signaling as a significant pathway in glutamatergic neurons (*P*_*adj*_ = 0.028; Supplementary Table 4b).

**Table 2:** Summary of pathway analysis (IPA) by brain cell type.

Cell Type	No. of analyzed DEGs^a^	Significant Canonical Pathways	Top 5 Enriched Pathways (IPA)
GABAergic neurons	121	Yes	Synaptogenesis Signaling Pathway, Neuropathic Pain Signaling in Dorsal Horn Neurons, Dopamine-DARPP32 Feedback in cAMP Signaling, Synaptic Long Term Depression, Circadian Rhythm Signaling
Glutamatergic neurons	316	Yes	Synaptogenesis Signaling Pathway, Synaptic Long Term Depression, Dopamine-DARPP32 Feedback in cAMP Signaling, Netrin Signaling, Neurovascular Coupling Signaling Pathway
Microglia	95	Yes	Protein Kinase A Signaling, Role of NFAT in Cardiac Hypertrophy, Cholecystokinin/Gastrin-mediated Signaling, Sperm motility, Cardiac Hypertrophy Signaling
Astrocytes	11	No	NA
Oligodendrocytes	39	No	NA
Polydendrocytes	63	No	NA
Endothelia	15	No	NA

*DEG* differential expressed genes, *IPA* Ingenuity Pathway Analysis.

^a^Analyzed DEGs correspond to genes with log2 fold changes < −1 or >1.

## Discussion

This study demonstrates that specific cell types within temporal neocortex contribute to memory outcomes in TLE. In fact, we observed marked heterogeneity between memory phenotypes with numerous differentially expressed genes across all brain cell types (i.e., astrocytes, endothelia, GABAergic and glutamatergic neurons, microglia, oligodendrocytes, and polydendrocytes).

Importantly, the most notable expression differences between those with and without impaired memory were observed in glutamatergic (excitatory) and GABAergic (inhibitory) neurons and included a number of genes related to neurodegenerative disorders and/or memory function (e.g., *APP*, *MAP2*, *MAPT*, *NEFL*, *PRKN*). Relatedly, the ‘Synaptogenesis Signaling Pathway’ was the top pathway for both neuronal types. Synaptogenesis, along with remodeling and growth of existing synapses, is known to play a critical role in learning and memory processes, including consolidation and long-term memory storage^10–12^. There is also evidence to suggest that aberrations in synaptic development and plasticity are associated with age-related memory decline, as well as memory and other cognitive impairments in psychiatric, neurological, and neurodegenerative disorders^10,13^. ‘Long-term potentiation’ and ‘synaptic depression,’ processes known to be essential for new memory formation, were also top pathways for both neuronal cell types^14–16^. While it was not surprising to identify DEGs relevant to neurons when we studied expression changes emanating from glutamatergic and GABAergic neurons, pathway analysis indeed helped put these findings into biological context.

These results demonstrate, using human temporal lobe brain tissue, that episodic memory impairment in temporal lobe epilepsy is related to molecular alterations within the temporal lobe and that these alterations are largely driven by changes in inhibitory (glutamatergic) and excitatory (GABAergic) neuronal cell populations. Importantly, these results also show that RNA expression differences at the single-nucleus level often show opposite directionality compared to findings in bulk RNA-seq data^9^, highlighting the unique contribution single-nucleus data can provide to our understanding of the brain transcriptome^17^. Specifically, in our prior bulk RNA-seq studies, we found the majority of DEGs were underexpressed in patients with impaired memory compared to those with intact memory^9^. In contrast, our snRNA-seq data show the majority of DEGs are overexpressed in the memory-impaired group compared to the memory-intact group. These findings are perhaps not surprising given recent transcriptomic work in the human brain demonstrating that bulk RNA is dominated by expression changes within excitatory neurons and oligodendrocytes – the most abundant cortical cell classes – and that changes in other cell types, particularly microglia, are not captured well with bulk RNA sequencing^18^. As a result, bulk RNA-seq analyses can miss DEGs with opposite directionality at the cell-type-specific level (i.e., *overexpressed* in one cell type and *underexpressed* in another)^18^. Additional studies will be required to examine this further and to replicate findings with a larger number of samples. Larger sample sizes will also permit use of mixed-effects models and/or pseudobulk analyses, which could not be employed here due to sample size limitations and the number of covariates. Future studies will also seek to determine whether similar molecular alterations are observed in the hippocampus of individuals with TLE with and without memory impairment and whether there are regional differences in transcript expression within specific subregions (e.g., cornu ammonis fields, dentate gyrus, subiculum) that contribute to memory impairment in TLE. These findings will challenge whether TLE and memory circuitry are governed by canonical or non-canonical pathways that are likely to cross-talk.

## Methods

### Participants

Fresh-frozen brain tissue samples were obtained from the temporal neocortex of 6 adults with pharmacoresistant TLE who underwent left (language-dominant) temporal lobe resections for treatment of their epilepsy and who completed comprehensive neuropsychological evaluations, including assessment of episodic memory, prior to surgery. Patients were 41 years of age on average (SD = 10) with 14 years of education (SD = 2). All patients self-identified as White, non-Hispanic, and half the sample was female. Mean age at seizure onset was 27 years (SD = 14), and mean duration of epilepsy was 14 years (SD = 8). Tissue specimens and clinical data were obtained from IRB-approved epilepsy data registries at the Cleveland Clinic, and the methods were performed in accordance with relevant guidelines and regulations approved by the Cleveland Clinic Institutional Review Board. All patients provided written informed consent.

### Episodic memory assessment

All patients completed measures of verbal episodic memory as part of preoperative neuropsychological evaluations a median of 6 months before surgery. Story recall was assessed with the Logical Memory subtest of the Wechsler Memory Scale – Third or Fourth Edition, and word-list learning was assessed with the Rey Auditory Verbal Learning Test. These measures were scored using demographically-corrected norms and transformed into standard scores (SS; mean = 100, SD = 15). Patients were separated into one of two memory phenotypes based on a mean composite delayed memory score (combined delayed story recall and word-list learning tasks). Specifically, mean scores <85 were classified as “impaired” memory (*n* = 4) and ≥85 were classified as “intact” memory (*n* = 2). As intended, memory scores were significantly lower in the impaired memory group (SS = 57) compared to the intact memory group (SS = 95).

### Tissue preparation and snRNA-Seq

Approximately 20 mg of fresh-frozen tissue (predominantly gray matter) from resected temporal neocortex was used from each patient to prepare the nuclei suspension. Our snRNA experiments were conducted using the “nuclei village” approach, a multiplex analysis of nuclei sampled from brain specimens from multiple donors simultaneously^19^. The nuclei were extracted, processed, and analyzed together, facilitating rigorous cross-sample comparisons. In subsequent computational analysis, combinations of hundreds of transcribed SNPs (for which alleles are ascertained in the RNA data) were used to assign each nucleus to the patient-of-origin. After treating the patient nuclei pool with myelin removal beads (Miltenyi Biotec, Bergisch Gladbach, Germany), 2X the standard number of 10X snRNA-Seq nuclei were loaded into 8 reactions (32,000 nuclei per reaction). The 8 reactions were sequenced on a NovaSeq S4 flow cell (Illumina, San Diego, CA, USA). After aligning to human reference GRCh38 with non-canonical contigs masked out, the libraries were run through CellBender to remove technical artifacts^20^. Then, high-quality cells were selected based on a combination of the number of unique molecular identifiers (UMIs, at least 400) and the percent of intronic reads (at least 40%). An average of 6486 nuclei per donor were identified. Nuclei from different samples were integrated and clustered using the Seurat package^21^. The clusters were then annotated using *scPred*^22^ and visualized with the *t*-SNE plots.

Differential expression analyses were conducted between subjects with impaired and intact memory per cell class, controlling for age, sex, and presence/absence of mesial temporal sclerosis, using the MAST package^23^. The following cell classes were examined: astrocytes, endothelia, GABAergic neurons, glutamatergic neurons, microglia, oligodendrocytes, and polydendrocytes. Pathway analyses were first performed with Kyoto Encyclopedia of Genes and Genomes (KEGG)^24^ using all DEGs. Then, a more stringent and comprehensive analysis, including only those DEGs with log2fold changes >1 or <−1, was performed with Ingenuity Pathway Analyses (IPA, QIAGEN). Enrichment for diseases and functions was also examined using IPA.

### Statistical analyses

Baseline descriptive statistics stratified by memory group (intact versus impaired) were calculated. Independent samples t-tests or Fisher’s exact tests were used to examine group differences on demographic and disease-related variables. *P* values of <0.05 were considered statistically significant. For differential expression analysis, pathway analysis and enrichment of diseases and functions, adjusted *P* values of <0.05 were considered statistically significant.

### Reporting summary

Further information on research design is available in the Nature Research Reporting Summary linked to this article.

## Supplementary information

Supplementary Material

Supplementary Table 2

Supplementary Table 3

Supplementary Table 4

Supplementary Table 5

Reporting Summary

## Data Availability

The data that support the findings of this study are not publicly available because of IRB-based restricted access. Sharing of data with a qualified researcher may be permissible with IRB-approval and a data use agreement. Further information about the datasets is available from the corresponding author on reasonable request.

## Code availability

The code used for data analyses is available upon request.
