# Supplementary material for: Brain single cell transcriptomic profiles in episodic memory phenotypes associated with temporal lobe epilepsy
Source: NPJ Genom Med. 2022 Nov 29;7:69. doi: 10.1038/s41525-022-00339-4 (PMC9709106; doi:10.1038/s41525-022-00339-4)
Supplement: Supplementary file 1 — Supplementary Material [file 41525_2022_339_MOESM1_ESM.docx]

**Supplementary Material**

**Brain Single Cell Transcriptomic Profiles in Episodic Memory Phenotypes Associated with Temporal Lobe Epilepsy**

Robyn M. Busch, Ph.D., Lamis Yehia, Ph.D., Bo Hu, Ph.D., Melissa Goldman, B.S., Bruce P. Hermann, Ph.D., Imad M. Najm, M.D., Steven A. McCarroll, Ph.D. & Charis Eng, M.D., Ph.D.

**Table of Contents**

Supplementary [Figure 1: t-SNE plot demonstrating clusters of nuclei by cell type and by patient. t-distributed stochastic neighbor embedding (t-SNE) plots of nuclei from temporal lobe tissue of patients with and without memory impairment a) by cell type and b) by patient. 2](#_Toc96415955)

Supplementary [Table 1: Number of genes and UMIs by cluster 3](#_Toc96415956)

Supplementary [Table 2: Differentially expressed genes between patients with and without impaired memory (see Excel files)](#_Toc96415957)

***Supplementary Table 2a - in GABAergic neurons***

***Supplementary Table 2b - in glutamatergic neurons***

***Supplementary Table 2c - in microglia***

***Supplementary Table 2d - in astrocytes***

***Supplementary Table 2e - in oligodendrocytes***

***Supplementary Table 2f - in polydendrocytes***

***Supplementary Table 2g - in endothelia***

Supplementary [Table 3: Pathway enrichment analyses using KEGG (see Excel files)](#_Toc96415957)

***Supplementary Table 3a - in GABAergic neurons***

***Supplementary Table 3b - in glutamatergic neurons***

***Supplementary Table 3c - in astrocytes***

***Supplementary Table 3d - in oligodendrocytes***

***Supplementary Table 3e - in polydendrocytes***

Supplementary [Table 4: Pathway enrichment analyses using IPA (see Excel files)](#_Toc96415957)

***Supplementary Table 4a - in GABAergic neurons***

***Supplementary Table 4b - in glutamatergic neurons***

Supplementary [Table 5: Diseases and functions using IPA (see Excel files)](#_Toc96415957)

***Supplementary Table 5a - in GABAergic neurons***

***Supplementary Table 5b - in glutamatergic neurons***

**Supplementary Figure 1.**

^
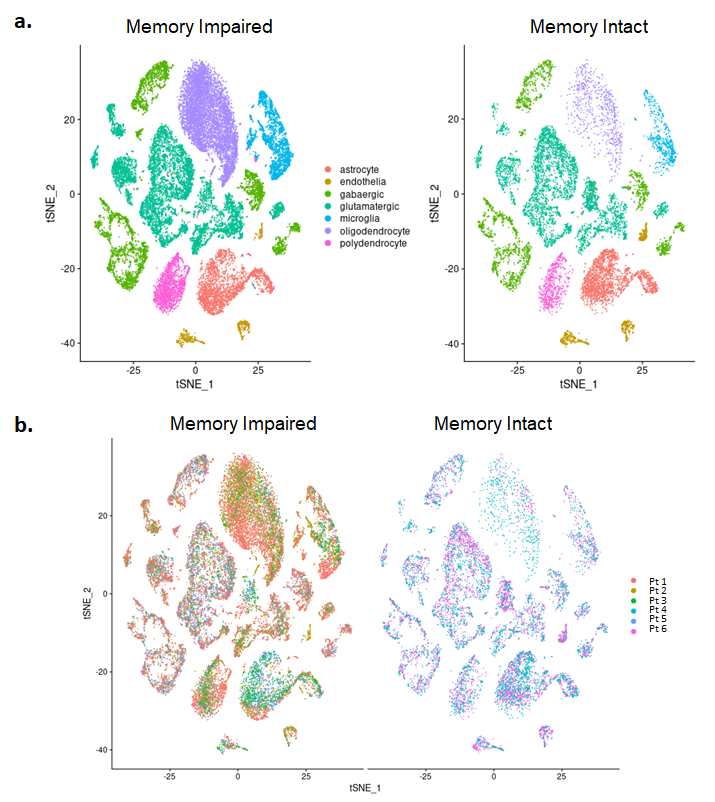
^

**Supplementary Table 1.**

|  | **Number of Genes**  Median (IQR) | **Number of UMIs**  Median (IQR) |
| --- | --- | --- |
| **astrocyte** | 4106 (3259, 5543) | 2221 (1875, 2682) |
| **endothelia** | 1880 (1312, 2890) | 1217 (920, 1638) |
| **GABAergic** | 7773 (5385, 12188) | 3565 (2872, 4496) |
| **glutamatergic** | 12600 (7830, 21194) | 4736 (3685, 6008) |
| **microglia** | 2182 (1436, 3458) | 1318 (976, 1897) |
| **oligodendrocyte** | 2593 (1875, 3456) | 1421 (1136, 1749) |
| **polydendrocyte** | 3842 (2548, 5535) | 2035 (1579, 2570) |

*Abbreviations*: UMI, unique molecular identifier; IQR, interquartile range
