# Supplementary material for: Brain single cell transcriptomic profiles in episodic memory phenotypes associated with temporal lobe epilepsy
Source: NPJ Genom Med. 2022 Nov 29;7:69. doi: 10.1038/s41525-022-00339-4 (PMC9709106; doi:10.1038/s41525-022-00339-4)
Supplement: Supplementary file 6 — Reporting Summary [file 41525_2022_339_MOESM6_ESM.pdf]

nature portfolio

Corresponding author(s): Robyn M Busch

Last updated by author(s): Oct 31, 2022

Reporting Summary

Nature Portfolio wishes to improve the reproducibility of the work that we publish. This form provides structure for consistency and transparency in reporting. For further information on Nature Portfolio policies, see our Editorial Policies and the Editorial Policy Checklist.

Statistics

For all statistical analyses, confirm that the following items are present in the figure legend, table legend, main text, or Methods section.

n/a	Confirmed	
☐	☒	The exact sample size (*n*) for each experimental group/condition, given as a discrete number and unit of measurement
☐	☒	A statement on whether measurements were taken from distinct samples or whether the same sample was measured repeatedly
☐	☒	The statistical test(s) used AND whether they are one- or two-sided<br>*Only common tests should be described solely by name; describe more complex techniques in the Methods section.*
☐	☒	A description of all covariates tested
☒	☐	A description of any assumptions or corrections, such as tests of normality and adjustment for multiple comparisons
☐	☒	A full description of the statistical parameters including central tendency (e.g. means) or other basic estimates (e.g. regression coefficient) AND variation (e.g. standard deviation) or associated estimates of uncertainty (e.g. confidence intervals)
☒	☐	For null hypothesis testing, the test statistic (e.g. *F*, *t*, *r*) with confidence intervals, effect sizes, degrees of freedom and *P* value noted<br>*Give P values as exact values whenever suitable.*
☒	☐	For Bayesian analysis, information on the choice of priors and Markov chain Monte Carlo settings
☒	☐	For hierarchical and complex designs, identification of the appropriate level for tests and full reporting of outcomes
☒	☐	Estimates of effect sizes (e.g. Cohen's *d*, Pearson's *r*), indicating how they were calculated

Our web collection on statistics for biologists contains articles on many of the points above.

Software and code

Policy information about availability of computer code

Data collection	After aligning to human reference GRCh38 with non-canonical contigs masked out, libraries were run through CellBender to remove technical artifacts. High-quality cells were selected based on a combination of the number of unique molecular identifiers (UMIs, at least 400) and the percent of intronic reads (at least 40%). An average of 6,486 nuclei per donor were identified. Nuclei from different samples were integrated and clustered using the Seurat package.
Data analysis	Clusters were annotated using scPred and visualized with the t-SNE plots. Pathway analyses were performed using Kyoto Encyclopedia of Genes and Genomes (KEGG) and Ingenuity Pathway Analyses (IPA, QIAGEN).

For manuscripts utilizing custom algorithms or software that are central to the research but not yet described in published literature, software must be made available to editors and reviewers. We strongly encourage code deposition in a community repository (e.g. GitHub). See the Nature Portfolio guidelines for submitting code & software for further information.

Data

Policy information about availability of data

All manuscripts must include a data availability statement. This statement should provide the following information, where applicable:

- Accession codes, unique identifiers, or web links for publicly available datasets
- A description of any restrictions on data availability
- For clinical datasets or third party data, please ensure that the statement adheres to our policy

The data that support the findings of this study are not publicly available because of IRB-based restricted access, but further information about the datasets is available from the corresponding author on reasonable request.

Human research participants

Policy information about studies involving human research participants and Sex and Gender in Research.

Reporting on sex and gender	Information regarding participant sex was included and the study groups were well balanced on this variable. Sex was also included as a covariate in all analyses.
Population characteristics	See below.
Recruitment	Participants were individuals who underwent epilepsy surgery at Cleveland Clinic and who had consented to have their data included in one of our Epilepsy research registries.
Ethics oversight	Cleveland Clinic Institutional Review Board

Note that full information on the approval of the study protocol must also be provided in the manuscript.

Field-specific reporting

Please select the one below that is the best fit for your research. If you are not sure, read the appropriate sections before making your selection.

☐ Life sciences ☒ Behavioural & social sciences ☐ Ecological, evolutionary & environmental sciences

For a reference copy of the document with all sections, see nature.com/documents/nr-reporting-summary-flat.pdf

Behavioural & social sciences study design

All studies must disclose on these points even when the disclosure is negative.

Study description	quantitative cross-sectional
Research sample	participant data and samples were obtained from existing IRB approved data registries at Cleveland Clinic
Sampling strategy	participant data and samples were obtained from existing IRB approved data registries at Cleveland Clinic
Data collection	participant data and samples were obtained from existing IRB approved data registries at Cleveland Clinic
Timing	data for this study were obtained from existing IRB approved data registries for individuals who have undergone epilepsy surgery at Cleveland Clinic
Data exclusions	no data were excluded
Non-participation	not applicable
Randomization	participants were allocated to groups based on their memory performance (impaired vs. intact)

Reporting for specific materials, systems and methods

We require information from authors about some types of materials, experimental systems and methods used in many studies. Here, indicate whether each material, system or method listed is relevant to your study. If you are not sure if a list item applies to your research, read the appropriate section before selecting a response.

Materials & experimental systems

n/a	Involved in the study	
☒	☐	Antibodies
☒	☐	Eukaryotic cell lines
☒	☐	Palaeontology and archaeology
☒	☐	Animals and other organisms
☐	☒	Clinical data
☒	☐	Dual use research of concern

Methods

n/a	Involved in the study	
☒	☐	ChIP-seq
☒	☐	Flow cytometry
☒	☐	MRI-based neuroimaging

Clinical data

Policy information about clinical studies

All manuscripts should comply with the ICMJE guidelines for publication of clinical research and a completed CONSORT checklist must be included with all submissions.

Clinical trial registration	not applicable
Study protocol	not applicable
Data collection	data and samples were collected at Cleveland Clinic (Cleveland, OH) from individuals who underwent epilepsy surgery and who consented to participation in an IRB-approved biorepository/registry
Outcomes	participants were allocated to groups based on their memory performance (impaired vs. intact)
